# The Sterility of Allotriploid Fish and Fertility of Female Autotriploid Fish

**DOI:** 10.3389/fgene.2019.00377

**Published:** 2019-04-26

**Authors:** Fangzhou Hu, Jingjing Fan, Qinbo Qin, Yangyang Huo, Yude Wang, Chang Wu, Qingfeng Liu, Wuhui Li, Xuan Chen, Cao Liu, Min Tao, Shi Wang, Rurong Zhao, Kaikun Luo, Shaojun Liu

**Affiliations:** State Key Laboratory of Developmental Biology of Freshwater Fish, College of Life Science, Hunan Normal University, Changsha, China

**Keywords:** distant hybridization, autotriploid, allotriploid, fertility, sterility

## Abstract

Based on the formation of an autotetraploid fish line (4nAUT, 4n = 200; F_2_–F_11_) derived from the distant hybridization of female *Carassius auratus* red var. (RCC, 2n = 100) × male *Megalobrama amblycephala* (BSB, 2n = 48), we produced autotriploid hybrids (3nAUT) by crossing females of RCC with males of 4nAUT and allotriploid hybrids (3nALT) by crossing females of *Cyprinus carpio* (CC, 2n = 100) with males of 4nAUT. The aim of this study was to comparatively investigate the reproductive characteristics of 3nALT and 3nAUT. We investigated morphological traits, chromosomal numbers, DNA content and gonadal development in 3nAUT and 3nALT. The results indicated both 3nAUT and 3nALT possessed 150 chromosomes and were triploid hybrids. The females and males of 3nALT and males of 3nAUT had abnormal gonadal development and could not generate mature eggs or sperm, but the females of 3nAUT had normal gonadal development and generated mature eggs at 2 years old. The females of 3nAUT generated different sizes of eggs, which fertilized with haploid sperm from RCC and formed viable diploid, triploid, and tetraploid offspring. The formation of these two kinds of triploid hybrids provides an ideal model for studying the reproductive traits of triploid hybrids, which is of great value in animal genetics and reproductive biology.

## Introduction

Polyploids are organisms that normally have three or more chromosome sets. Polyploidy is common in plants, and studies have shown that all angiosperms are ancient polyploids (Otto, 2007). As research continues, increasing evidence has shown that polyploids are also widespread in animals and are mainly concentrated in amphibians, reptiles, and fishes (Mable, 2004; Gregory and Mable, 2005; Wertheim et al., 2013). Polyploids can be divided into autopolyploids and allopolyploids according to their origin of chromosome doubling. Allopolyploids possess a combination of chromosomes from two or more different species, while autopolyploids possess multiple chromosome sets mainly derived from a single taxon.

As lower vertebrates, fish chromosomes display plasticity and thus produce polyploids more easily (Liu, 2010). Triploid fish are found spontaneously in both wild and cultured populations and can be induced via physical or chemical methods. The artificial induction of triploid fish is mainly used to improve quality associated with sexual maturation such as higher growth rates, stronger disease resistance, and better organoleptic properties (Cuñado et al., 2002; Cal et al., 2005;Poontawee et al., 2007; Maxime, 2008; Werner et al., 2008; Chen et al., 2009; Kavumpurath and Pandian, 2010).

Fish eggs are released at the metaphase stage of meiosis II. Further resumption of meiosis II of the eggs is induced by the entry of the spermatozoon (Colas and Dubé, 1998). Thus, physical or chemical treatments applied during meiosis II can prevent the extrusion of the second polar body while allowing chromosomal division, thus producing triploids (Piferrer et al., 2009). Generally, physical and chemical treatments are successfully used to induce triploidy in many fishes (Chourrout, 1984, 1988; Haffray et al., 2007; Xu et al., 2010). However, the survival of triploids is usually very low due to the physical and chemical treatments damaging the fertilized eggs. Triploid fish can also be mass produced using indirect methods based on distant hybridization (Biradar and Rayburn, 1993; Bullini, 1994; Mallet, 2007; Liu, 2010; Hu et al., 2018). In our previous study, both females and males of fertile allotetraploid fish (4n = 200) were produced by crossing female red crucian carp and male common carp (Liu et al., 2001). Sterile triploids have been produced at a large scale by crossing allotetraploid and diploid fish (Liu et al., 2001; Chen et al., 2009).

Additionally, in our previous study, we successfully produced both females and males of fertile allotetraploid hybrids (F_1_, 4n = 148) in the first generation of *Carassius auratus* red var. (2n = 100) × *Megalobrama amblycephala* (2n = 48) (Liu et al., 2007). Due to the abnormal chromosome behavior during meiosis of F_1_ hybrids, autodiploid sperm and autodiploid ova were produced and used to fertilize each other, finally resulting in the formation of autotetraploid F_2_. Surprisingly, the females and males of autotetraploids could produce diploid eggs and diploid spermatozoa, respectively. These diploid gametes could be fertilized to form the next generation of autotetraploid fish. The F_2_–F_11_ of the autotetraploid stocks have been established in succession (Qin et al., 2014). In the present study, based on the formation of 4nAUT, we successfully obtained autotriploid hybrids (3nAUT) and allotriploid hybrids (3nALT) by crossing female RCC × male 4nAUT (F_10_) and female *Cyprinus carpio* (CC, 2n = 100) × male 4nAUT (F_10_), respectively. Furthermore, we investigated important biological traits of 3nAUT and 3nALT, including morphological traits, chromosomal numbers, DNA content and gonadal development. This study is of importance for fish genetic breeding and fish reproductive biology.

## Materials and Methods

### Animals and Crosses

RCC, CC, 4nAUT (F_10_), 3nAUT, and 3nALT were obtained from the Protection Station of Polyploid Fish at Hunan Normal University. During the reproductive seasons (from April to June each year), 20 mature female RCC and CC and male 4nAUT (F_10_) were chosen as the parents. The crossings were performed in two groups. In the first group, RCC was used as the maternal line and 4nAUT was used as the paternal line. In the second group, CC was used as the maternal line and 4nAUT was used as the paternal line. The mature eggs and sperm of RCC (CC) and 4nAUT were fertilized and the embryos developed in culture dishes at a water temperature of 20–22°C. In each group, 5000 embryos were taken at random for the examination of fertilization rate (number of embryos at the gastrula stage/number of eggs) and the hatching rate (number of hatched fry/number of eggs). The hatched fry were transferred to a pond for further culture.

### Morphological Traits

At 1 year of age, 20 RCC, 20 CC, 20 4nAUT, 20 3nAUT, and 20 3nALT were randomly selected for morphological examination following the methods described in a previous study (Hu et al., 2012). For both measurable and countable data, we used the software SPSS 22.0 to analyze the covariance of the data between hybrid offspring and their parents.

### Measurement of DNA Content

The DNA content of erythrocytes of RCC, CC, 4nAUT and their hybrid offspring was measured using a flow cytometer (cell counter analyzer, Partec). Approximately 0.5–1 ml of red blood cells was collected from the caudal vein of the above fish into syringes containing 100–200 units of sodium heparin. The blood samples were treated following the method described in a previous paper (Liu et al., 2001). The DNA content of each sample was measured under the same conditions. To calculate the probabilities of the ratios of the DNA content of the polyploid hybrids to the sum of that of RCC (CC) and 4nAUT, the x^2^ test with Yate’s correction was used for testing deviation from expected ratio values.

### Preparation of Chromosome Spreads

To determine ploidy, chromosomal preparations were performed from peripheral blood cell cultures of 20 3nAUT and 20 3nALT at 1 year of age. The chromosomes were prepared in accordance with a previous study (Liu et al., 2001). First, about 0.1 ml blood was collected from each sample using a syringe soaked with 0.1% sodium heparin, cultured in nutrient solution at 25.5°C and 5% CO_2_ for 72 h, then colchicine was added 3.5 h before harvest. Cells were harvested by centrifugation, followed by hypotonic treatment with 0.075 M KCl at 26°C for 30 min, then fixed in methanol–acetic acid (3:1, v/v) with three changes. Cells were dropped onto cold slides, air-dried and stained for 30 min in 4% Giemsa solution. The shape and number of chromosomes were analyzed under a microscope. In total, 100 metaphase spreads (50 metaphase spreads for each sample) of chromosomes were analyzed.

### Gonadal Structures and Gamete Phenotypes

At ages of 1 and 2 years, 50 3nAUT and 50 3nALT individuals were randomly sampled for examination of gonad development via histological sectioning. The gonads were fixed in Bouin’s solution, embedded in paraffin, sectioned, and stained with hematoxylin and eosin. Gonadal structures were observed and photographed with a Pixera Pro 600ES digital camera (Nikon, Japan). The gonadal stages were classified in accordance with a prior standard series for cyprinid fish (Sun et al., 2003). In addition, at 2 years old, the mature eggs or water-like semen were squeezed out from the females and males of 3nAUT, respectively. The mature eggs and semen were collected for morphological examination.

### Egg Ploidy Detection

The 2-year-old female 3nAUT could produce different-sized eggs. To determine egg ploidy, mature eggs were used to fertilize RCC haploid sperm and viable offspring were produced. The ploidy of these hybrid offspring was determined by flow cytometric analysis of DNA content in erythrocytes.

## Results

### The Formation of Two Triploid Hybrids

**FIGURE 1:**
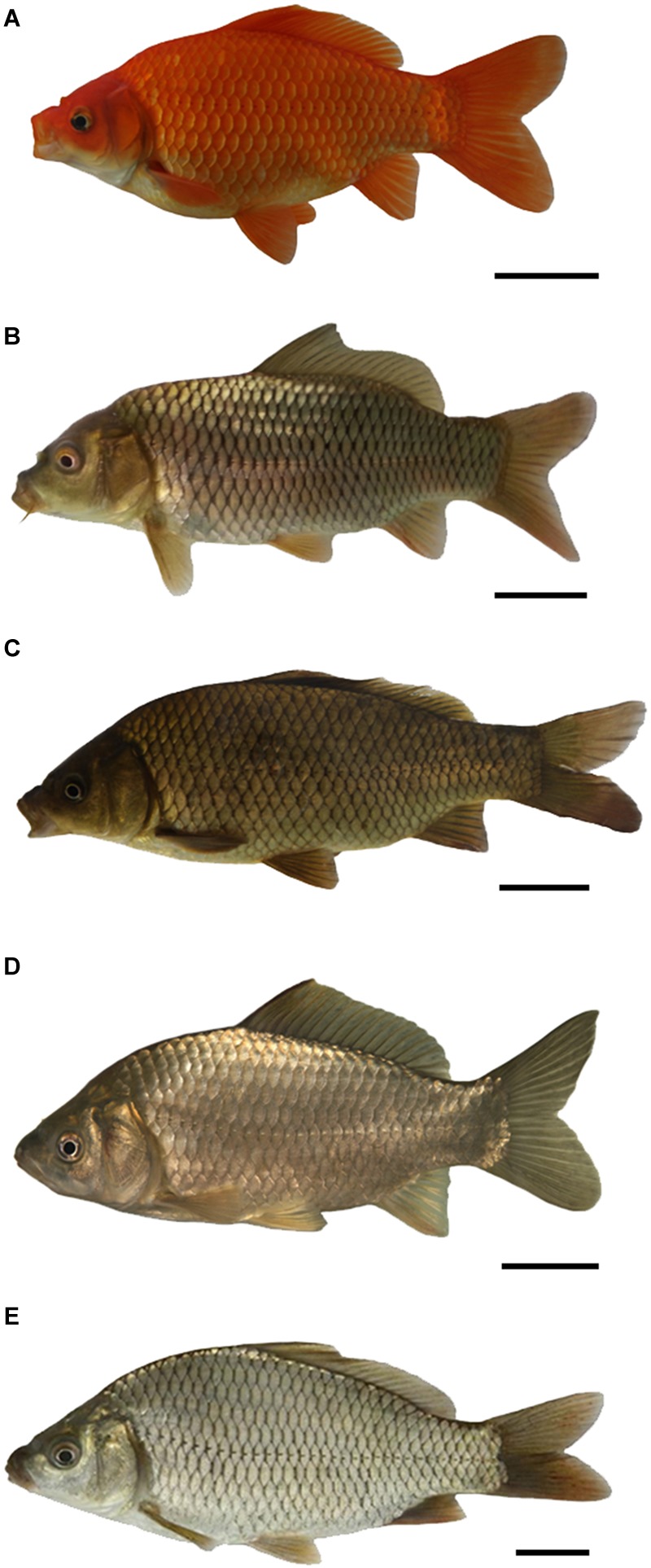
Appearance of RCC, CC, 4nAUT, 3nAUT, and 3nALT. **(A)** RCC; **(B)** CC; **(C)** 4nAUT; **(D)** 3nAUT; **(E)** 3nALT. Bar = 5 cm.

During the reproductive season (from April to June), 3nAUT (Figure 1D) were produced by crossing female RCC (Figure 1A) and male 4nAUT (Figure 1C). 3nALT (Figure 1E) were produced by crossing female CC (Figure 1B) and male 4nAUT (Figure 1C). A high fertilization rate (>96.4%) and hatch rate (>86.7%) were observed in both groups (Table 1).

**Table 1:** Fertilization rate and hatch rate of the two groups.

Hybridization groups	Fertilization rate (%)	Hatch rate (%)
RCC × 4nAUT	97.8	86.7
CC × 4nAUT	96.4	88.8

### Phenotypes of Hybrids and Their Parents

The phenotypes of RCC, 4nAUT, CC, 3nALT, and 3nAUT are illustrated in Figure 1. The counts traits and measurable traits of RCC, 4nAUT, CC, 3nALT, and 3nAUT are shown in Tables 2, 3. Several morphological differences were detected both between 3nALT and their parental, 3nAUT and their parental (Tables 2, 3). In addition, the main morphological differences between 3nALT and 3nAUT are that 3nALT has two pairs of barbels and 33–34 lateral scales, whereas the 3nAUT have no barbels and 30–31 lateral scales (Figure 1 and Table 2).

**Table 2:** Comparison of the countable traits between the hybrid offspring and their parents.

Fish type	No. of lateral scales	No. of upper lateral scales	No. of lower lateral scales	No. of dorsal fins	No. of abdominal fins	No. of anal fins
RCC	29.20 ± 0.51^bcde^ (28–30)	5.60 ± 0.50^bcde^ (5–6)	5.70 ± 0.47^bcde^ (5–6)	III + 18.65 ± 0.49^bcde^ (III + 18–19)	8.55 ± 0.51^ce^ (8–9)	III + 5.65 ± 0.49^bcde^ (III + 5–6)
CC	36.35 ± 1.24^acde^ (35–38)	5.37 ± 0.45^ad^ (5–6)	5.30 ± 0.43^acde^ (5∼6)	III + 17.62 ± 0.89^acde^ (III + 17–19)	8.58 ± 0.61^cd^ (8–9)	III + 6.37 ± 0.39^acde^ (III + 6–7)
4nAUT	29.54 ± 1.03^abde^ (29–32)	5.36 ± (0.50)^ad^ (6–7)	6.81 ± 0.75^abde^ (6–7)	III + 18.27 ± 0.46^abde^ (III + 18–19)	8.63 ± 0.50^abd^ (8–9)	III + 5.45 ± 0.52^abde^ (III + 5–6)
3nAUT	30.56 ± 1.38^abce^ (30–31)	5.44 ± 0.84^abce^ (5–6)	7.14 ± 0.35^abce^ (7–8)	III + 16.86 ± 0.54^abce^ (III + 15–17)	8.54 ± 0.30^bce^ (8–9)	III + 5.55 ± 0.78^abce^ (III + 6–7)
3nALT	33.64 ± 1.52^abcd^ (33–34)	5.33 ± 0.25^abd^ (5–6)	7.05 ± 0.88^abcd^ (7–8)	III + 15.78 ± 0.38^abcd^ (III + 15–16)	8.60 ± 0.32^ad^ (8–9)	III + 6.15 ± 0.68^abcd^ (III + 6–7)

*Values in the same column with letter a, b, c, d, e for each species show significant differences with RCC, CC, 4nAUT, 3nAUT, and 3nALT (*P* < 0.05).*

**Table 3:** Comparison of the measurable traits between the hybrid offspring and their parents.

Fish type	WL/BL	BL/BW	BL/HL	HL/HW	TL/TW	BW/HW
RCC	1.22 ± 0.02^b^	2.18 ± 0.02^bcde^	3.72 ± 0.03^bde^	1.07 ± 0.03^bde^	0.82 ± 0.03^bde^	1.84 ± 0.03^bcde^
CC	0.83 ± 0.07^a^	2.94 ± 0.01^acde^	4.17 ± 0.02^acde^	1.23 ± 0.07^acd^	1.16 ± 0.11^acde^	1.67 ± 0.01^acde^
4nAUT	1.23 ± 0.02^b^	2.23 ± 0.08^abde^	3.74 ± 0.07^bde^	1.08 ± 0.02^bde^	0.84 ± 0.02^be^	1.88 ± 0.06^abde^
3nAUT	1.24 ± 0.02^b^	2.48 ± 0.11^abce^	4.03 ± 0.58^abce^	0.98 ± 0.14^abce^	0.87 ± 0.05^abe^	1.57 ± 0.06^abce^
3nALT	1.22 ± 0.02^b^	2.67 ± 0.13^abcd^	4.36 ± 0.35^abcd^	1.24 ± 0.01^acd^	0.96 ± 0.08^abcd^	1.63 ± 0.13^abcd^

*Values in the same column with letter a, b, c, d, e for each species show significant differences with RCC, CC, 4nAUT, 3nAUT, and 3nALT (*P* < 0.05).*

### DNA Content of Two Triploid Hybrids and Their Parents

**FIGURE 2:**
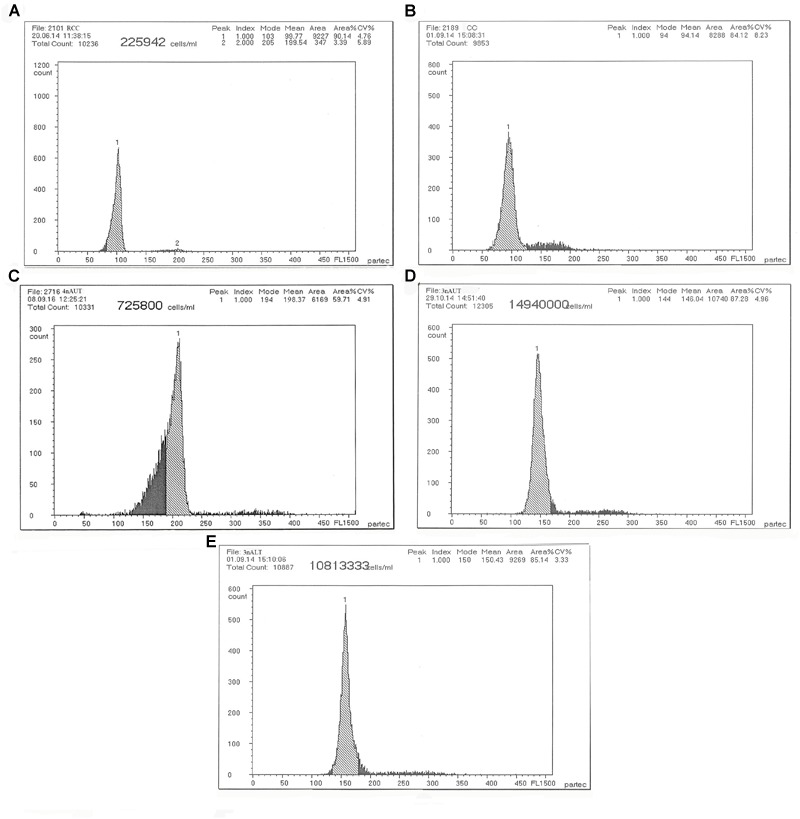
Cytometric histograms of DNA fluorescence for RCC, CC, 4nAUT, 3nAUT, and 3nALT. **(A)** The mean DNA content of RCC (peak 1: 99.77). **(B)** The mean DNA content of CC (peak 1: 94.14). **(C)** The mean DNA content of 4nAUT (peak 1: 198.37). **(D)** The mean DNA content of 3nAUT (peak 1: 146.04). **(E)** The mean DNA content of 3nALT (peak 1: 150.43).

The DNA content of the parents RCC, CC and 4nAUT were used as the controls (Figure 2 and Table 4). The results of the comparisons of DNA content between hybrids and their parents are shown in Table 4. The mean DNA content of 3nALT and 3nAUT was equal (*P* > 0.05) to the sum of one parent and half of the other parent, indicating that they were triploids (Figure 2).

**Table 4:** Mean DNA content of 3nAUT, 3nALT and their parents.

Fish type	DNA content	Ratio	
		Observed	Expected
RCC	99.77		
CC	94.14		
4nAUT	198.37		
3nALT	150.43	(4nAUT+CC)/3nALT = 1.94^a^	2
3nAUT	146.04	(4nAUT+RCC)/3nAUT = 2.04^a^	2

*^*a*^The observed ratio was not significantly different (*P* > 0.05) from the expected ratio.*

### Chromosome Number of Two Triploid Hybrids and Their Parents

**FIGURE 3:**
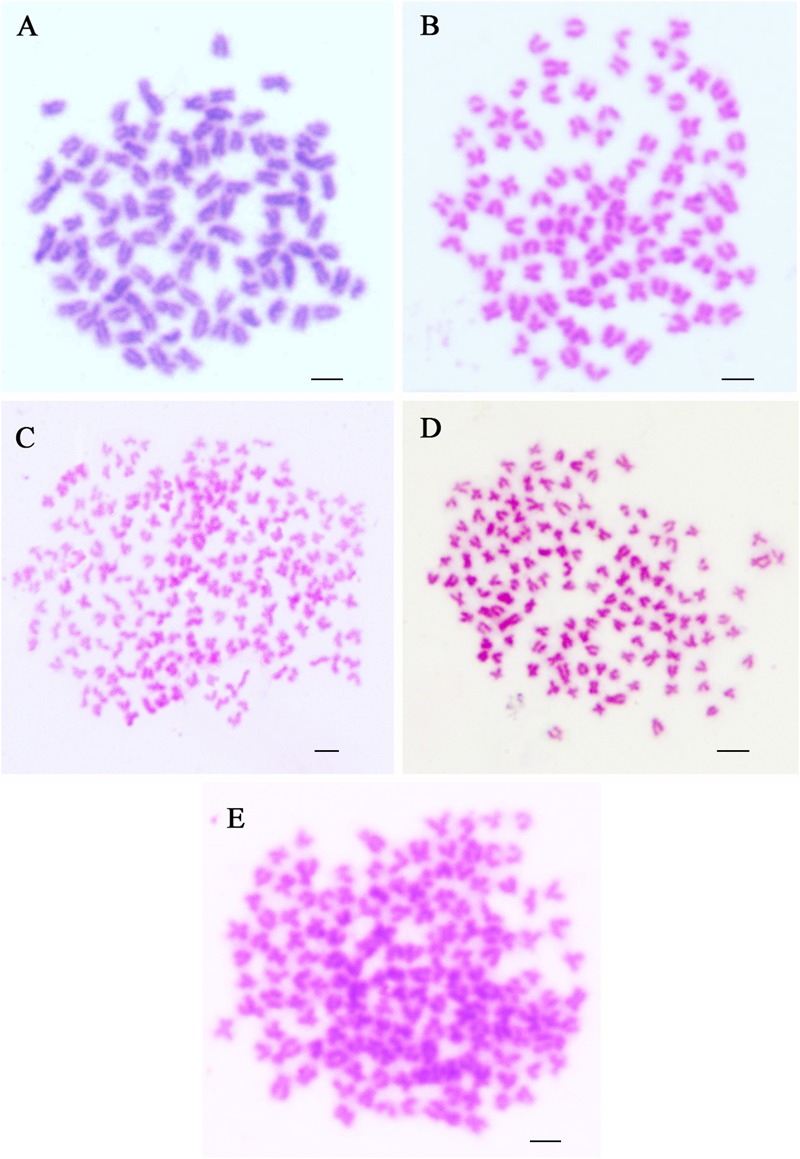
Chromosome spreads at metaphase in RCC, CC, 4nAUT, 3nAUT, and 3nALT. **(A)** The metaphase chromosome spreads of RCC (2n = 100). **(B)** Metaphase chromosome spreads of CC (2n = 100). **(C)** Metaphase chromosome spreads of 4nAUT (4n = 200). **(D)** Metaphase chromosome spreads of 3nAUT (3n = 150). **(E)** Metaphase chromosome spreads of 3nALT (3n = 150). Bar in **(A–E)**, 4 μm.

Chromosomes were counted in 10 metaphase spreads for each sample of RCC, CC, 4nAUT, 3nALT, and 3nAUT (Figure 3 and Table 5). For RCC, 92.5% of chromosomal metaphases possessed 100 chromosomes, indicating that they were diploids with 100 chromosomes (2n = 100) (Figure 3A and Table 5). For CC, 95.5% of chromosomal metaphases had 100 chromosomes, indicating they were diploids with 100 chromosomes (2n = 100) (Figure 3B and Table 5). For 4nAUT, 81.0% of chromosomal metaphases had 200 chromosomes, indicating they were tetraploid with 200 chromosomes (4n = 200) (Figure 3C and Table 5). For 3nAUT, 88.0% of chromosomal metaphases had 150 chromosomes, indicating they were triploid with 150 chromosomes (4n = 200) (Figure 3D and Table 5). For 3nALT, 89.5% of chromosomal metaphases had 150 chromosomes, indicating they were triploid with 150 chromosomes (4n = 200) (Figure 3E and Table 5).

**Table 5:** Examination of chromosome number in 3nAUT, 3nALT and their parents.

Fish type	No. in metaphase	Distribution of chromosome number					
		<100	100	<150	150	<200	200
RCC	200	15	185				
CC	200	9	191				
4nAUT	200					38	162
3nAUT	200			24	176		
3nALT	200			21	179		

### Fertility of the Two Types of Triploid Hybrids

**FIGURE 4:**
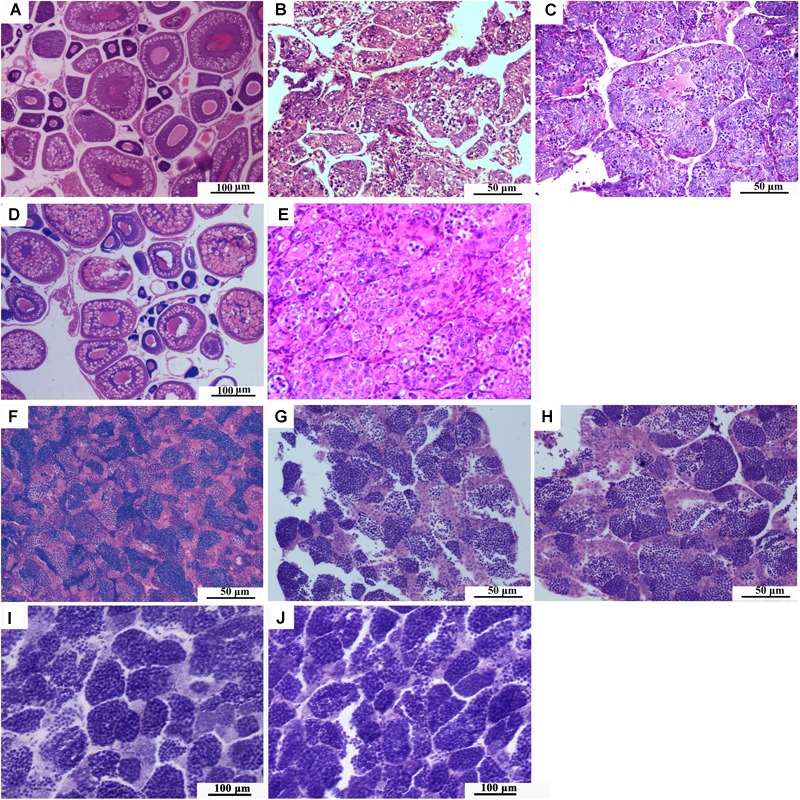
Gonadal development. **(A)** Histological section of ovary of 1-year-old RCC. **(B)** Histological section of ovary of 1-year-old 3nALT. **(C)** Histological section of ovary of 1-year-old 3nAUT. **(D)** Histological section of ovary of 2-year-old 3nAUT. **(E)** Histological section of ovary of 2-year-old 3nALT. **(F)** Histological section of testis of 1-year-old RCC. **(G)** Histological section of testis of 1-year-old 3nAUT. **(H)** Histological section of testis of 1-year-old 3nALT. **(I)** Histological section of testis of 2-year-old 3nAUT. **(J)** Histological section of testis of 2-year-old 3nALT.

The ovaries of 1-year-old RCC developed well and contained stages II, III, and IV oocytes (Figure 4A). The testes of 1-year-old RCC contained many lobules in which there were many mature spermatozoa and spermatids (Figure 4F).

The ovaries of 1-year-old 3nALT contained many oogonium-like cells but very few ova at stage II (Figure 4B). In the testes of 1-year-old 3nALT, some spermatogonia developed into primary spermatocytes (Figure 4G). In the ovaries of 2-year-old 3nALT, the oogonium-like cells were disintegrating (Figure 4E). In 2-year-old male 3nALT, a number of empty seminiferous tubules lacking secondary spermatocytes or sperm were observed in the testes (Figure 4J). In the reproductive season, no milt or eggs were stripped out from the 2-year old males and females of 3nALT. These results suggest that 3nALT were sterile.

**FIGURE 5:**
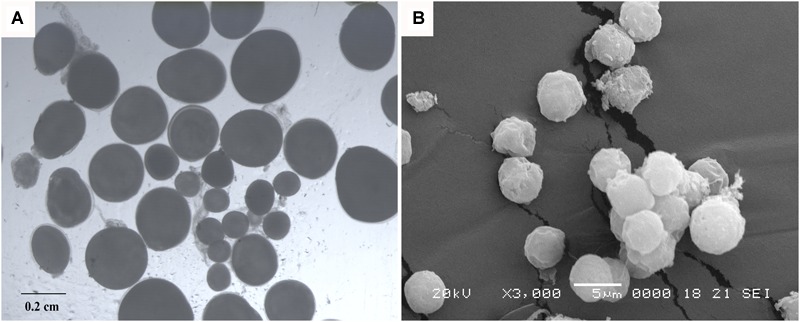
Eggs and spermatozoa of 3nAUT. **(A)** Different sizes of eggs were collected from 2-year-old females of 3nAUT. **(B)** Abnormal spermatozoa produced by males of 3nAUT.

The ovaries of 1-year-old 3nAUT were partially developed. Many oogonia proliferated massively with a few having developed into oocytes of phase II (Figure 4C). In the testes of 1-year-old 3nAUT, some spermatogonia developed into primary spermatocytes (Figure 4F), but no semen could be squeezed out of the testes. The ovaries of 2-year-old 3nAUT developed well and contained stages II, III, and IV oocytes (Figure 4D). The testes of 2-year-old 3nAUT contained blunt spermatid-like cells, many spermatogonia with heteromorphous and cavitate nuclei or with a few sperms that lacked tails or nuclei (Figure 4I). In the reproductive season, water-like semen and different sizes of eggs were collected from 2-year-old males and females of 3nAUT, respectively (Figure 5).

### Egg Ploidy

**FIGURE 6:**
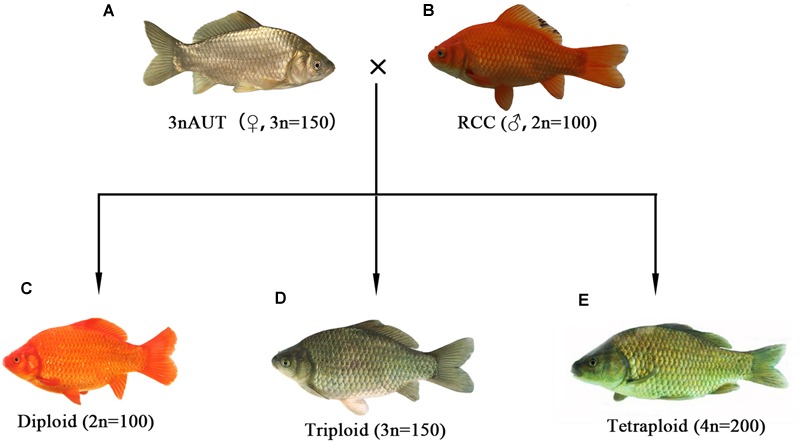
Formation of the polyploid hybrids of female 3nAUT × male RCC. **(A)** Female 3nAUT; **(B)** male RCC; **(C)** diploid offspring; **(D)** triploid offspring; **(E)** tetraploid offspring.

The ploidy levels of the crossing offspring of female 3nAUT and male RCC were confirmed by measuring DNA content. The results show that diploid, triploid, and tetraploid hybrid were successfully obtained by crossing female 3nAUT and male RCC (Figure 6). These results indicate that female 3nAUT produce eggs of at least three different ploidy levels, including haploid, diploid, and triploid eggs.

## Discussion

Distant hybridization is an important means of fish genetic breeding and is also an effective way to produce polyploid offspring. In our previous study, autotetraploid hybrid lines were established from the distant hybridization of red crucian carp × blunt snout bream (*Megalobrama amblycephala*) (Liu et al., 2007; Qin et al., 2014). In the present study, 3nAUT and 3nALT were produced by crossing female RCC × male 4nAUT and female *Cyprinus carpio* (CC, 2n = 100) × male 4nAUT, respectively (Figure 1).

Distant hybridization is a useful strategy to produce hybrid offspring with altered genotypes (Bullini, 1994; Liu et al., 2007; Hu et al., 2018). Compared with their parents, obvious differences were found in 3nALT and 3nAUT in the measurable and countable data, indicating the distant hybridizing effect (Figure 1). Additionally, most of the countable and measurable traits were significantly different between 3nAUT and 3nALT (*P* < 0.05) (Tables 2, 3). It was easy to distinguish 3nAUT and 3nALT, as 3nALT have two short barbels and 3nAUT have no barbels (Figure 1).

Examining the DNA content is a rapid and simple method of determining the ploidy of samples. Counting the chromosomal number is a direct and accurate method. In this study, the ploidy levels of 3nAUT and 3nALT were confirmed by measuring DNA content (Figure 2 and Table 4) and counting chromosomal number (Figure 3 and Table 4). All of the above results were in agreement that both 3nAUT and 3nALT were triploid hybrids.

In aquaculture, induced triploidy is mainly used for the production of sterile fish. According to traditional concepts, triploid fish usually have disordered meiosis, which can lead to low fertility or complete infertility (Vrijenhoek, 1994, 2006; Liu et al., 2000; Yin et al., 2000; Peter et al., 2010). In allotriploid fish, functional sterility may reflect genomic imbalances due to the presence of an extra set of chromosomes (Krisfalusi et al., 2000). Infertile allotriploid fish have been reported in some studies (Liu et al., 2007; He et al., 2013; Xiao et al., 2014; Hu et al., 2018). In autotriploid fish, meiosis is seriously impacted because three sets homologous chromosomes cannot correctly pair during the zygotene stage of prophase I (Carrasco et al., 1998; Cuñado et al., 2002). In the present study, the gonadal development of both 3nAUT and 3nALT was examined by means of microscopic tissue sections. The results show that 3nALT were sterile and that their gonadal development was abnormal (Figure 4). Male 3nAUT also could not produce normal sperm and was sterile. But, female 3nAUT were fertile and could produce different-sized eggs during the reproductive seasons. Han et al. (2010) reported infertility of female triploid rainbow trout caused by developmental abortion of oocytes and that oogonia formed cytocysts before the prophase oocytes. A similar result was found in female triploid yellowtail tetra (Ferreira et al., 2017). In contrast, Gomelsky et al. (2016) described a fertile triploid female koi, which could produce aneuploid eggs. Similarly, in our study, female 3nAUT could produce eggs with at least three different ploidy levels. Further study will be needed to determine whether there are aneuploid eggs.

In general, half-reduced gametes are produced by meiosis in animals. For example, diploid fish usually produce haploid gametes. However, there are some reports of the production of unreduced gametes generated by hybrids. For example, female triploid loach produced haploid and triploid eggs (Zhang et al., 1998). The female and male diploid hybrids of koi *Cyprinus carpio* × goldfish *Carassius auratus* produced unreduced diploid eggs and diploid sperm, respectively (Delomas et al., 2017). In our previous study, we found that the female allotetraploid hybrids produced diploid and tetraploid eggs (Liu et al., 2007; Hu et al., 2018). The formation of these unreduced gametes may be related to premeiotic endoreduplication, endomitosis or fusion of germ cells (Allen and Stanley, 1978; Yoshikawa et al., 2008; Liu, 2010). In this study, the 3N eggs produced by 3nAUT may be due to premeiotic endoreduplication of oogonia, thereby forming 6N oogonia and ultimately forming 3N eggs. Additionally, the formation of 1N and 2N eggs may relate to abnormal behavior of chromosomes during meiosis in female 3nAUT. This phenomenon is most common in autotriploid plants (Lange and Wagenvoort, 1973; Del Bosco et al., 2007). Interestingly, though they had the same parents, female 3nAUT were fertile, while males were sterile. This difference may exist because some genes that control meiosis have sex-specific expression (Kaul and Murthy, 1985; Maceira et al., 1992).

In summary, the formation of both 3nALT and 3nAUT is of great value in aquaculture and fisheries. The sterility of 3nALT ensures that it is unable to mate with other wild fish, and this would play an important role in protecting wild fish resources. Besides, 3nAUT can be used as a model to research productive rules of distant hybrid polyploid progeny, and fertile female 3nAUT also provide a special resource for fish breeding.

## Ethics Statement

All the fish were cultured in ponds at the Protection Station of Polyploid Fish, Hunan Normal University. Fish treatments were performed according to the Care and Use of Agricultural Animals in Agricultural Research and Teaching. This study was approved by the Science and Technology Bureau of China. Approval from the Department of Wildlife Administration was not required for the experiments conducted in this study. Before dissection, fish were deeply anesthetized with 100 mg/L MS-222 (Sigma-Aldrich).

## Author Contributions

SL contributed to the conception and design of the study. FH, WL, QL, XC, YW, CL, and YH performed the experimental work. SL and FH participated in drafting the manuscript. JF, CW, QQ, and MT analyzed the data. SW, RZ, and KL participated in interpretation and discussion of the results. All authors read and approved the final manuscript.

## Conflict of Interest Statement

The authors declare that the research was conducted in the absence of any commercial or financial relationships that could be construed as a potential conflict of interest.
